# Spotlight on Early COVID-19 Research Productivity: A 1-Year Bibliometric Analysis

**DOI:** 10.3389/fpubh.2022.811885

**Published:** 2022-05-31

**Authors:** Panagiotis Giannos, Konstantinos S. Kechagias, Konstantinos Katsikas Triantafyllidis, Matthew E. Falagas

**Affiliations:** ^1^Society of Meta-Research and Biomedical Innovation, London, United Kingdom; ^2^Department of Life Sciences, Faculty of Natural Sciences, Imperial College London, London, United Kingdom; ^3^Department of Metabolism, Digestion and Reproduction, Faculty of Medicine, Imperial College London, London, United Kingdom; ^4^Department of Dietetics, West Suffolk Hospital NHS Foundation Trust, Bury St Edmunds, United Kingdom; ^5^Alfa Institute of Biomedical Sciences, Athens, Greece

**Keywords:** coronavirus, COVID-19, SARS-CoV-2, bibliometrics, scientometrics, research productivity, collaborations, international

## Abstract

Coronavirus disease 2019 (COVID-19), one of the most serious public health crises in over a century, has led to an unprecedented surge of publications across all areas of knowledge. This study assessed the early research productivity on COVID-19 in terms of vaccination, diagnosis, treatment, symptoms, risk factors, nutrition, and economy. The Scopus database was searched between January 1, 2020 and December 31, 2020 to initially examine the research productivity on COVID-19, as measured by total publications by the 20 highest-ranked countries according to gross domestic product. The literature search was then refined, and research productivity was assessed across seven major research domains related to COVID-19: vaccination, diagnosis, treatment, symptoms, risk factors, nutrition, and economy. The initial literature search yielded 53,348 publications. Among these, 27,801 publications involved authorship from a single country and 22,119 publications involved authorship from multiple countries. Overall, the United States was the most productive country (*n* = 13,491), with one and a half times or more publications than any other country, on COVID-19 and the selected domains related to it. However, following adjustment for population size, gross domestic product, and expenditure for research and development, countries of emerging economies such as India along countries of lower population density such as Switzerland, Indonesia, and Turkey exhibited higher research productivity. The surge of COVID-19 publications in such a short period of time underlines the capacity of the scientific community to respond against a global health emergency; however where future research priorities and resource distribution should be placed on the respective thematic fields at an international level, warrants further investigation.

## Introduction

In December 2019, 44 cases of viral pneumonia with a characteristic clinical presentation of fever, dry cough, dyspnoea, and severe progressive respiratory distress were reported for the first time in Wuhan, Hubei Province of China (1–3). In January 2020, the Chinese Center for Disease Control and Prevention announced that the etiology of this atypical pneumonia was a novel coronavirus which was then named severe acute respiratory syndrome coronavirus 2 (SARS-CoV-2) (4–6). The disease caused by SARS-CoV2 (COVID-19) was initially declared as a Public Health Emergency of International Concern by the WHO, and in March of 2020, it was classified as a pandemic (7).

As in the two preceding outbreaks of coronavirus disease-severe acute respiratory syndrome (SARS) in 2002 and Middle East respiratory syndrome (MERS) in (i.e. 2012, COVID-19) has placed significant pressure to public health structures and caused economic implications worldwide (8, 9). In less than a year, SARS-CoV-2 has spread in more than 190 countries and has resulted in over 76 million confirmed cases with more than 1.7 million deaths (10). Additionally, the global reaction with pandemic mitigation measures has caused a major disruption in economic activity, resulting in the worst recession since the Great Depression (11).

In response to this global health emergency, the COVID-19 pandemic has also triggered an unprecedented response from the scientific community (12). The global dissemination of this disease has sparked an explosive increase in scientific output across various research domains to address the unmet need for its control (13–16).

Bibliometric analyses, which examine the contribution of countries to a specific research field of the literature by focusing on the statistical appraisal of scholarly output, have gained much attention (17, 18). This is attributed to their ability in predicting research trends across the current and emerging thematic areas and guide the focus of the global literature. Bibliometric studies have been widely applied at multiple scholarly areas with few successfully guiding decision-making across the respective thematic fields (19–21). At present, a plethora of scientometric and bibliometric studies have been published aiming at gaining more insights on the landscape of publications related to COVID-19 (13, 22–26). However, only a small portion of bibliometric analyses have explored temporally the pandemic in terms of research output during the first months (27–30), and additionally, the economic aspect driving scholarly productivity has not been systematically examined.

Aiming to fill this gap in the literature and guide future research priorities and resource distribution, we undertook a bibliometric exploration of the literature and assessed the early research productivity on COVID-19 and seven major research domains related to it by the 20 highest-ranked countries according to gross domestic product (GDP).

## Materials and Methods

We performed a comprehensive literature search of the Scopus database for publications on COVID-19 from the 20 highest-ranked countries according to GDP (United States, China, United Kingdom, Italy, India, Canada, Australia, Germany, Spain, France, Brazil, Turkey, Netherlands, Switzerland, Saudi Arabia, Japan, South Korea, Mexico, Indonesia, and Russian Federation), published between January 1, 2020 and December 31, 2020. The literature search was performed on a single day (January 12, 2021) to minimize the bias from daily database updates. Publications were limited to peer-reviewed original articles or review articles written in English. The language criterion was applied to ensure that the quality assessment of manuscripts was based on similar processing standards. The search of the literature was ensued by PG and KKT and extracted by KSK and PG.

From the initial literature search, we extracted a list of publications that included search terms in their title, abstract, or keywords which best describe COVID-19 terminology, namely “COVID-19” OR “SARS-COV2” OR “Coronavirus” OR “2019-nCoV”. We then performed seven further literature searches focusing on seven major research domains related to COVID-19, namely vaccination, diagnosis, treatment, symptoms, risk factors, nutrition, and economy. From these searches, we extracted a list of publications that included the following search terms in their title, abstract or keywords for the seven research domains: (1) vaccination: “(AND Vaccin^*^)”, (2) diagnosis: “(AND (Diagnos^*^ OR Test^*^))”, (3) treatment: “(AND (Treat^*^ OR Therap^*^ OR Pharma^*^ OR Drug^*^))”, (4) nutrition: “(AND (Nutri^*^ OR Diet^*^ OR Food^*^ OR Supplement^*^))”, (5) risk factors: “(AND ((Risk^*^ AND Factor^*^) OR (Predispos^*^ AND Factor^*^)))”, (6) symptoms: “(AND (Sign^*^ OR Symptom^*^ OR (Clinical AND Present^*^) OR Manifestation^*^))”, (7) economy “(AND (Econom^*^ OR Financ^*^ OR Wealth^*^ OR Trad^*^))” (Supplementary Table S1).

The resulting publications were grouped by country based on the country of origin mentioned in the affiliation of the first author. This was applicable to all publications even when these had multiple authors. Lastly, publications which had authorship from multiple countries were classified as collaborations.

The raw number of total publications and collaborations (overall and for each of the seven research domains) from each country was then normalized based on average population size, mean GDP, and percentage of GDP for research and development (R&D). This information was based on 2019 figures, or on the latest available data, and was extracted using the World Bank database.

## Results

**Figure 1:**
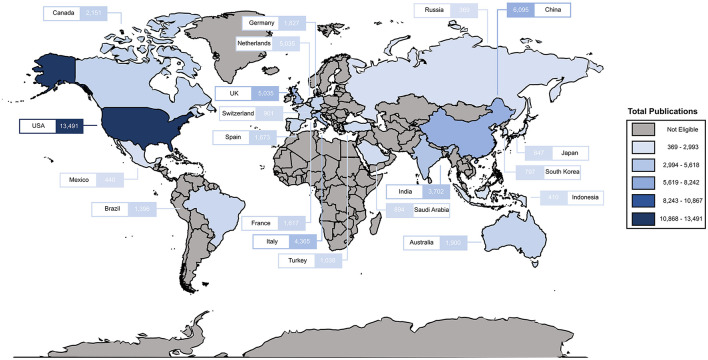
The world map of early research productivity on COVID-19 by the 20 highest-ranked countries according to gross domestic product.

**Figure 2:**
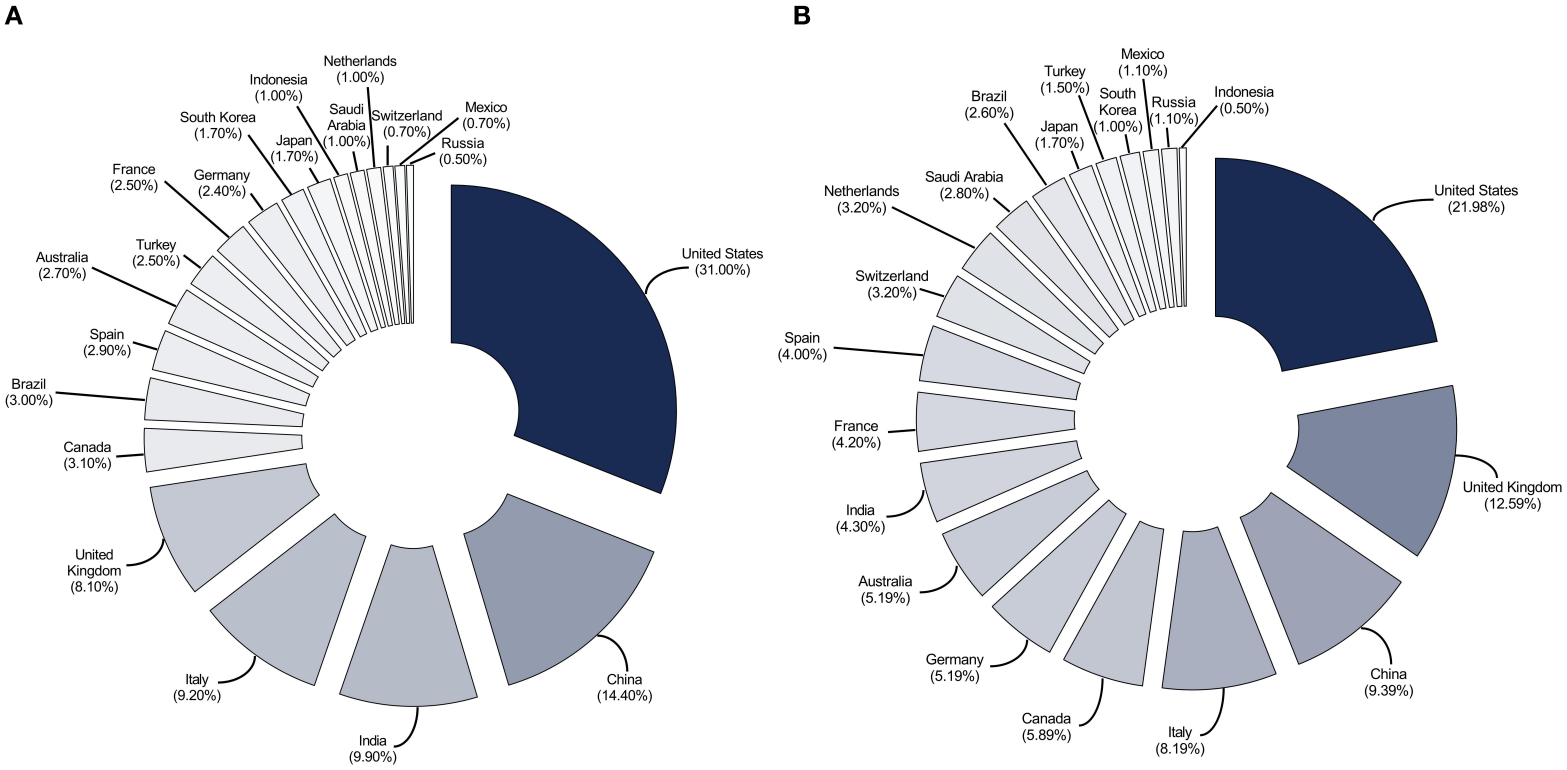
The proportion of early publications involving authorship from a single country **(A)** and multiple countries **(B)** on COVID-19 from the 20 highest-ranked countries according to gross domestic product.

The initial literature search using only COVID-19-specific terms yielded 53,348 publications that met the inclusion criteria. The search was further filtered based on the 20 highest-ranked countries according to GDP, and 49,920 total publications were retrieved (Figure 1). Among these, 27,801 publications involved authorship from a single country and 22,119 publications involved authorship from multiple countries (classed as collaborations) (Figure 2). Overall, the United States had the highest number of total publications, regardless of authorship from single or multiple countries. China and the United Kingdom were observed as the remaining in the three highest-ranked countries.

**Figure 3:**
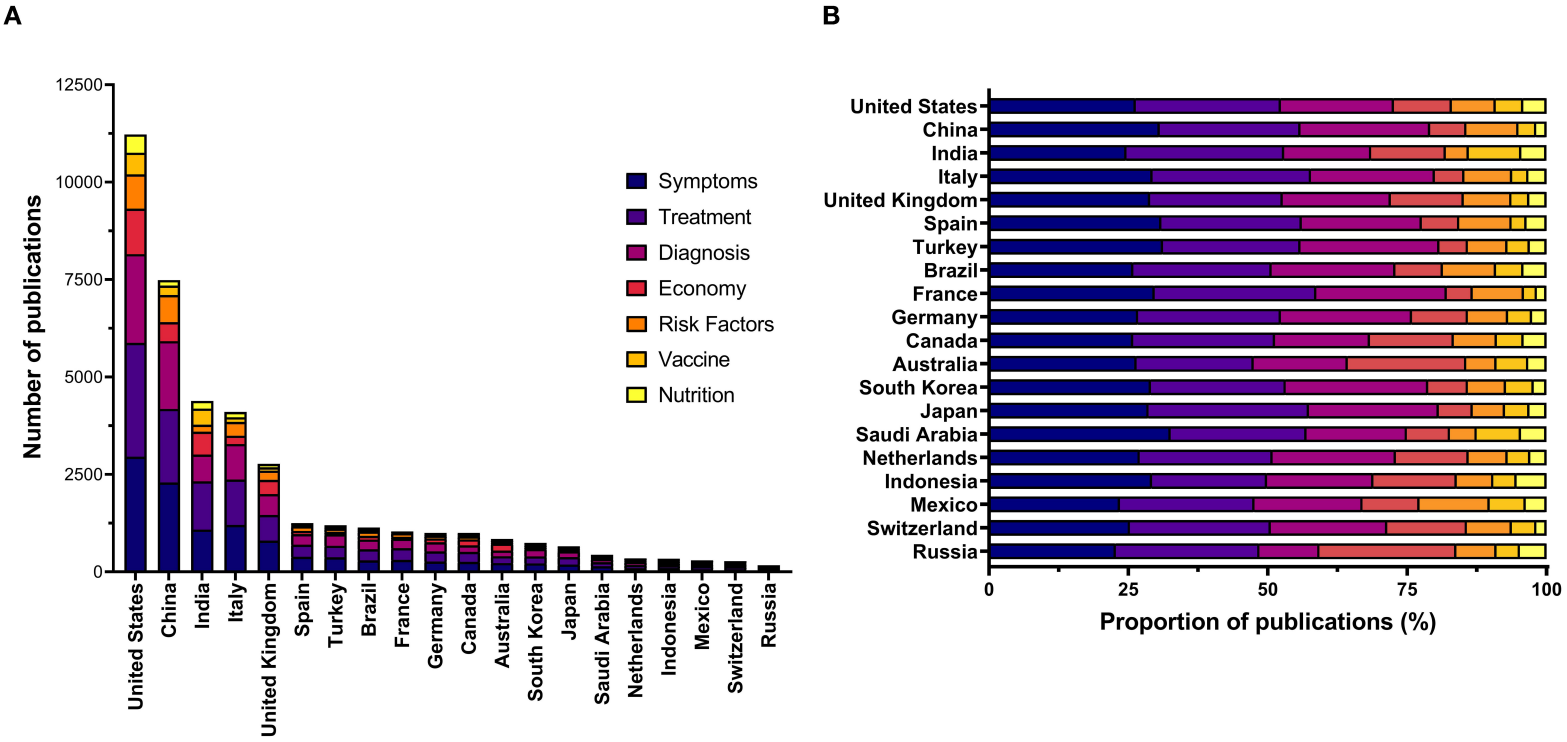
Total number **(A)** and proportion **(B)** of early publications involving authorship from a single country on COVID-19 from the 20 highest-ranked countries according to gross domestic product.

**Figure 4:**
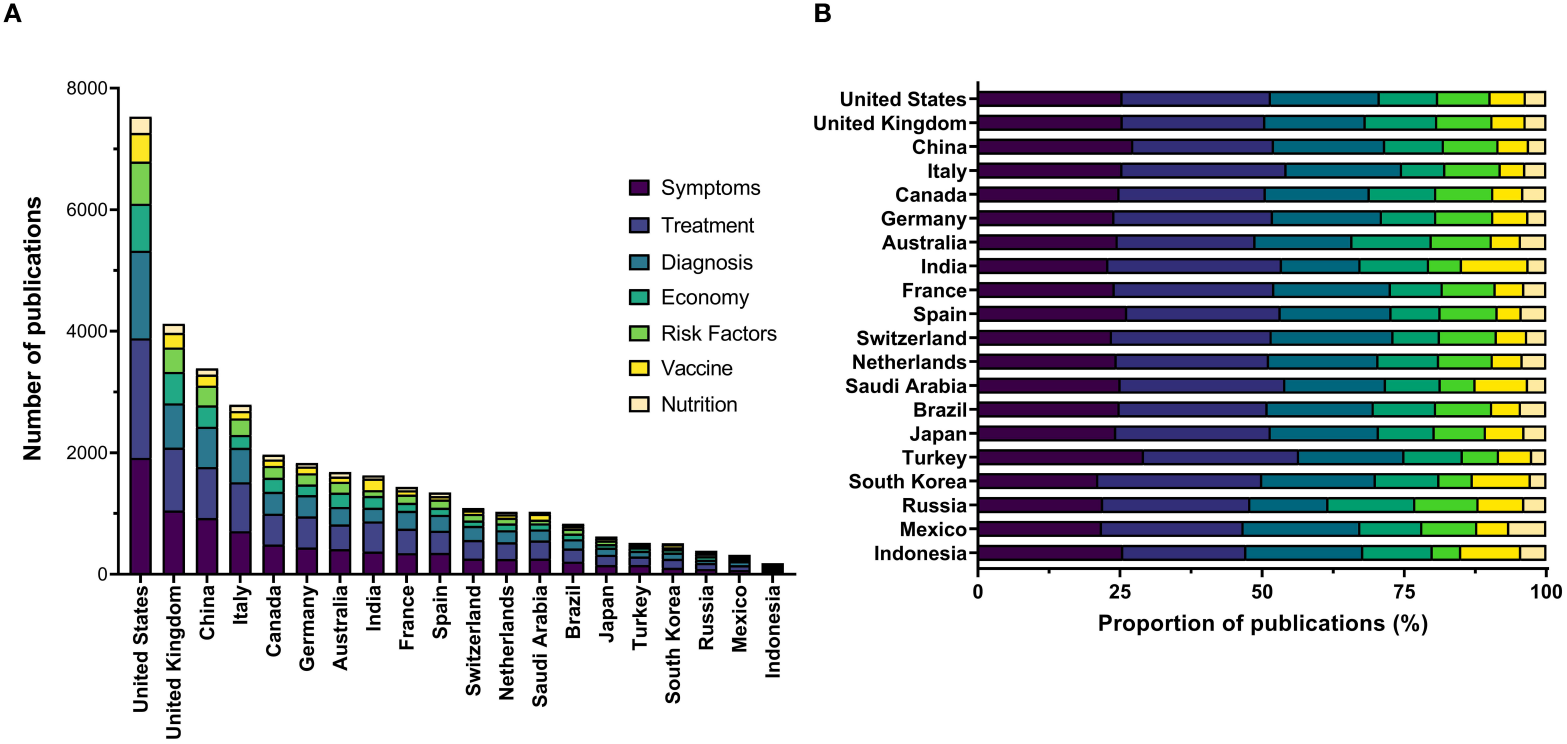
Total number **(A)** and proportion **(B)** of early publications involving authorships from multiple countries on COVID-19 from the 20 highest-ranked countries according to gross domestic product.

The literature was additionally assessed based on seven major research domains related to COVID-19 (vaccination, diagnosis, treatment, symptoms, risk factors, nutrition, and economy). We observed that the United States dominated across all the selected domains. China, India, Italy, and the United Kingdom were noted as the remaining highest-ranked countries in the aforementioned domains (Table 1, Figures 3, 4).

**Table 1:** Total number of early publications on selected research domains related to COVID-19 from the 20 highest-ranked countries according to GDP.

	**Vaccination**	**Diagnosis**	**Treatment**	**Nutrition**	**Risk Factors**	**Symptoms**	**Economy**
United States	1,025	3,718	4,887	740	1,575	4,870	1,942
China	421	2,402	2,727	243	1,023	3,218	838
Japan	71	271	358	44	94	339	101
Germany	156	586	767	84	256	707	275
India	604	910	1,737	249	276	1,455	783
United Kingdom	330	1,265	1,693	236	638	1,851	881
France	96	537	704	74	229	653	179
Italy	239	1,478	1,972	238	624	1,911	428
Brazil	98	407	498	84	190	501	188
Canada	152	530	761	121	274	748	380
Russian Federation	38	71	143	23	55	123	100
South Korea	89	292	327	31	81	324	110
Spain	90	530	677	103	252	738	200
Australia	134	430	585	103	224	638	415
Mexico	37	123	151	32	68	139	65
Indonesia	33	101	108	26	31	144	72
Netherlands	68	274	358	53	121	344	155
Saudi Arabia	130	261	405	53	84	401	133
Turkey	78	394	436	49	118	524	114
Switzerland	70	290	375	42	131	326	128
Totals	3,959	14,870	19,669	2,628	6,344	19,954	7,487

*GDP, gross domestic product*.

*Estimates of GDP were calculated based on 2019 figures, or on latest available data*.

When the number of total publications was normalized by population, Switzerland ranked highest, followed by the United Kingdom and Australia, while upon GDP, Italy scored highest, followed by the United Kingdom and Turkey. Lastly, when normalized by expenditure on R&D, India dominated, followed by Indonesia and Italy (Table 2).

**Table 2:** Adjusted early research productivity on COVID-19 from the 20 highest-ranked countries according to GDP.

	**Average population (thousand)**	**Average GDP (US$ million)**	**R&D expenditure (% GDP)**	**Gross domestic spending on R&D (US$ million)**	**Total publications during the study period**	**Total publications per population (thousand)**	**Total publications per GDP (US$ million)**	**Total publications per spending on R&D (US$ million)**
United States	328,239.52	21,374,418.88	2.84	607,033.50	13,491	4.11	0.06	2.22
China	1,397,715.00	14,342,902.84	2.19	314,109.57	6,095	0.44	0.04	1.94
Japan	12,6264.93	5,081,769.54	3.26	165,665.69	847	0.67	0.02	0.51
Germany	83,132.80	3,845,630.03	3.09	118,829.97	1,827	2.20	0.05	1.54
India	1,366,417.75	2,875,142.31	0.65	18,688.43	3,702	0.27	0.13	19.81
United Kingdom	66,834.40	2,827,113.18	1.72	48,626.35	5,035	7.53	0.18	10.35
France	67,059.89	2,715,518.27	2.20	59,741.40	1,617	2.41	0.06	2.71
Italy	60,297.40	2,001,244.39	1.40	28,017.42	4,365	7.24	0.22	15.58
Brazil	211,049.53	1,839,758.04	1.26	23,180.95	1,396	0.66	0.08	6.02
Canada	37,589.26	1,736,425.63	1.57	27,261.88	2,151	5.72	0.12	7.89
Russian Federation	144,373.54	1,699,876.58	0.99	16,828.78	369	0.26	0.02	2.19
South Korea	51,709.10	1,642,383.22	4.81	78,998.63	797	1.54	0.05	1.01
Spain	47,076.78	1,394,116.31	1.24	17,287.04	1,673	3.55	0.12	9.68
Australia	25,364.31	1,392,680.59	1.87	26,043.13	1,900	7.49	0.14	7.30
Mexico	127,575.53	1,258,286.72	0.31	3,900.69	440	0.34	0.03	11.28
Indonesia	270,625.57	1,119,190.78	0.23	2,574.14	410	0.15	0.04	15.93
Netherlands	17,332.85	909,070.40	2.16	19,635.92	972	5.61	0.11	4.95
Saudi Arabia	34,268.53	792,966.84	0.82	6,502.33	894	2.61	0.11	13.75
Turkey	83,429.62	754,411.71	0.96	7,242.35	1,038	1.24	0.14	14.33
Switzerland	8,574.83	703,082.44	3.37	23,693.88	901	10.51	0.13	3.80

*Population size, GDP and R&D expenditure estimates were calculated based on 2019 figures, or on latest available data*.

*GDP, gross domestic product; R&D, research and development*.

## Discussion

Our bibliometric study assessed the early research productivity on COVID-19 and seven major research domains related to it (vaccination, diagnosis, treatment, symptoms, risk factors, nutrition, and economy) by the 20 highest-ranked countries according to GDP. Our findings indicated that the United States had the highest number of total publications on COVID-19 and across all seven research domains related to it. However, after normalization based on average population size, mean GDP, and percentage of GDP for R&D, Switzerland, Italy, and India dominated in the number of total publications on COVID-19.

Our findings showed that the United States exhibited the highest research productivity and, thus, possibly the greatest contribution in the early scientific progress on COVID-19, responding with publications of extensive scope at an exponentially expanding rate. Concurrently, we also revealed that the United States embraced vastly global efforts against COVID-19, engaging highly with inter-institutional and international collaborations, displaying the highest research productivity in terms of collaborations. Taken together, these findings support that the United States might have adopted an early evidence-based response to reduce the impact of COVID-19 and are closely aligned with the country's expenditure on R&D, which is the largest worldwide.

A trend of increased overall research productivity on COVID-19 from two of the BRICS countries (China and India), Italy and the United Kingdom, was observed. BRICS countries have rapidly emerging economies with a constant increase in their average GDP and total health spending per capita since 1980 and 1995, respectively (31). Furthermore, the United Kingdom ranks highest worldwide in the field-weighted citation impact since 2007, a measure of research impact and quality (32). Lastly, an improved performance in Italy's research system has been documented when considering the resources allocated for research, ranking one of the highest in Europe based on number of publications for R&D expenditure (33). Although plausible, these potential explanations become questionable when considering that Italy, India, and the United Kingdom were in the 90^th^ percentile of countries with confirmed COVID-19 cases and deaths worldwide at the time of the study. In fact, Italy and the United Kingdom were also ranked on the 50^th^ percentile in terms of GDP of the included countries, while India was ranked on the latter 25^th^ percentile. On the contrary, China was the second highest-ranked in terms of GDP, with confirmed COVID-19 cases and deaths in the 60^th^ and 78^th^ percentiles, respectively (Organization, 2020). Thus, only China's performance could be reflective of the country's research capacity and its leading role in the early response to the disease.

After normalization average population size, mean GDP, and percentage of GDP for R&D, Switzerland, Italy, Indonesia, Turkey, and the United Kingdom exhibited the highest research productivity on COVID-19. Apart from India, Italy, and the United Kingdom which had a leading role against COVID-19 even prior adjustment, countries of lower population density such as Switzerland in Europe and Indonesia and Turkey in Asia rather predominated. In total, these results initially indicate that countries such as Switzerland, Italy, India, Indonesia, and Turkey may have also had strong contribution in the early scientific progress on COVID-19. Thus, our study highlights the impetuous by these countries to compete in R&D against the long-standing research-intensive countries such as the United States, China, and the United Kingdom. Overall, the observed regional distribution in research productivity on COVID-19 and the selected domains related to it may, in reality, be relevant to the context of the epidemic situation and a combination of other determinants such as the governmental policy response, the health infrastructure resilience, the research funding availability, and even the research workforce (34, 35).

This study makes a valuable contribution to the literature; however, a variety of limitations relevant to our search strategy, the filter criteria applied, and the protocol of data acquisition employed within the Scopus database exist. Initially, affiliation information of the retrieved publications did not particularly reflect the country in which the research was actually established or conducted, hinting an “overlap” bias in cases of publications with authorships from multiple countries. Although this is an inherent drawback in bibliometric studies, it has been demonstrated that even when a literature search is inflated by collaborative publications, the sensitivity of the search is not significantly affected (36). On the contrary, the filtering criteria applied to our search could have magnified its specificity and, thus, considerably decreased the breadth of suitable publications. In fact, as we adopted a search protocol with a focus on a single literature database (i.e., Scopus) rather than multiple ones (i.e., PubMed and Web of Science), this could have restricted the quantity of eligible publications. Moreover, we did not undertake manual screening of the retrieved publications and thus could not ensure that all the identified articles indeed focused on COVID-19 or the other research domains related to it. Lastly, a large proportion of suitable publications were available only in their original language, and by limiting suitable publications written only in English, we could have underestimated the research output of non-English-speaking countries or of those having non-English journals.

## Conclusions

The emergence of COVID-19 has generated an explosive increase in scientific production worldwide across all areas of knowledge, which underlies an unmet need for solutions against one of the most serious public health crises in over a century. Our findings ultimately demonstrated and confirmed a massive output in early COVID-19 publications thus far, higher than any previous outbreak. We identified that countries of emerging economies and of lower population density had increased overall research productivity along the long-standing research-intensive countries such as the United States, China, and the United Kingdom. This could provide insights as to which countries adopted an early evidence-based response to reduce the early impact of COVID-19, and where future research priorities and resource distribution should be placed on the respective thematic fields at an international level prospectively.

## Data Availability Statement

The original contributions presented in the study are included in the article/Supplementary Material, further inquiries can be directed to the corresponding authors.

## Author Contributions

PG, KSK, and KKT conceived and designed the study. PG and KKT acquired and collated the data. All authors contributed to the article and approved the submitted version.

## Conflict of Interest

The authors declare that the research was conducted in the absence of any commercial or financial relationships that could be construed as a potential conflict of interest.

## Publisher's Note

All claims expressed in this article are solely those of the authors and do not necessarily represent those of their affiliated organizations, or those of the publisher, the editors and the reviewers. Any product that may be evaluated in this article, or claim that may be made by its manufacturer, is not guaranteed or endorsed by the publisher.

## References

[1] Hui DS Azhar EE Madani TA Ntoumi F Kock R Dar O. The continuing epidemic threat of novel coronaviruses to global health–the latest novel coronavirus outbreak in Wuhan, China. Int J Infect Dis. (1920) 91:264–6. 10.1016/j.ijid.2020.01.009 PMC7128332 31953166

[2] Lu H Stratton CW Tang YW. Outbreak of pneumonia of unknown etiology in Wuhan, China: the mystery and the miracle. J Med Virol. (2020) 92:401–2. 10.1002/jmv.25678 31950516 PMC7166628

[3] Yang X Yu Y Xu J Shu H Liu H Wu Y. Clinical course and outcomes of critically ill patients with SARS-CoV-2 pneumonia in Wuhan, China: a single-centered, retrospective, observational study. Lancet Respir Med. (2020) 8:475–81. 10.1016/S2213-2600(20)30079-5 32105632 PMC7102538

[4] Li Q. An outbreak of NCIP (2019-nCoV) infection in China—wuhan, Hubei province, 2019–2020. China CDC Weekly. (2020) 2:79–80. 10.46234/ccdcw2020.022 34594812 PMC8393104

[5] Tan W Zhao X Ma X Wang W Niu P Xu W. A novel coronavirus genome identified in a cluster of pneumonia cases—Wuhan, China 2019–2020. China CDC Weekly. (2020) 2:61–2. 10.46234/ccdcw2020.017 34594763 PMC8393069

[6] Tavakoli A Vahdat K Keshavarz M. Novel coronavirus disease 2019 (COVID-19): an emerging infectious disease in the 21st century. ISMJ. (2020) 22:432–50. 10.29252/ismj.22.6.432

[7] Spinelli A Pellino G. COVID-19 pandemic: perspectives on an unfolding crisis. Br J Surg. (2020) 107:785–7. 10.1002/bjs.11627 32191340 PMC7228411

[8] Theocharis G Kechagias KS Oikonomou M Chorepsima S Rodis D Salpigktis I. A Model for Out-of-Hospital Multispecialty Emergency Medicine: Accomplishments and Challenges. Health Services Insights. (2018) 11:1178632918805996. 10.1177/1178632918805996 30369787 PMC6201181

[9] Fauci AS Lane HC Redfield RR. “Covid-19—navigating the uncharted”. Mass Medical Soc. (2020) 382:1268–9. 10.1056/NEJMe2002387 32109011 PMC7121221

[10] Dong E Hongru D Lauren G. An interactive web-based dashboard to track COVID-19 in real time. In: The Lancet Infectious Diseases. Lancet Publishing Group (2020) 20:533–4. 32087114 10.1016/S1473-3099(20)30120-1 PMC7159018

[11] Nicola M Alsafi Z Sohrabi C Kerwan A Al-Jabir A Iosifidis C. The socio-economic implications of the coronavirus and COVID-19 pandemic: a review. Int J Surg. (2020) 78:185–93. 10.1016/j.ijsu.2020.04.018 32305533 PMC7162753

[12] Putman MS Ruderman EM Niforatos JD. Publication rate and journal review time of COVID-19 related research. In: Mayo Clinic Proceedings: Mayo Foundation for Medical Education and Research. Elseiver (2020). 95:2290–1. 10.1016/j.mayocp.2020.08.017 33012362 PMC7458065

[13] Haghani M Bliemer MC Goerlandt F Li J. The scientific literature on Coronaviruses, COVID-19 and its associated safety-related research dimensions: a scientometric analysis and scoping review. Saf Sci. (2020) 129:104806. 10.1016/j.ssci.2020.104806 32382213 PMC7203062

[14] Triantafyllidis KK Giannos P Mian IT Kyrtsonis G Kechagias KS. Varicella zoster virus reactivation following COVID-19 vaccination: A systematic review of case reports. Vaccines. (2021) 9:1013. 10.3390/vaccines9091013 34579250 PMC8471236

[15] Giannos P Triantafyllidis KK Geropoulos G Kechagias KS. Persistent hiccups as an atypical presentation of SARS-CoV-2 infection: A systematic review of case reports. Front Neurol. (2022) 13:819624. 10.3389/fneur.2022.819624 35444608 PMC9014175

[16] Giannos P Prokopidis K. Gut dysbiosis and long COVID-19: Feeling gutted. J Med Virol. (2022). 10.1002/jmv.27684. [Epub ahead of print]. 35233795 PMC9088471

[17] Falagas ME Karavasiou AI Bliziotis IA. A bibliometric analysis of global trends of research productivity in tropical medicine. Acta Trop. (2006) 99:155–9. 10.1016/j.actatropica.2006.07.011 17014806

[18] Ellegaard O Wallin JA. The bibliometric analysis of scholarly production: How great is the impact? Scientometrics. (2015) 105:1809–31. 10.1007/s11192-015-1645-z 26594073 PMC4643120

[19] Lauer MS Danthi NS Kaltman J Wu C. Predicting productivity returns on investment: thirty years of peer review, grant funding, and publication of highly cited papers at the National Heart, Lung, and Blood Institute. Circ Res. (2015) 117:239–43. 10.1161/CIRCRESAHA.115.306830 26089369 PMC4506707

[20] Gal D Glänzel W Sipido KR. Mapping cross-border collaboration and communication in cardiovascular research from 1992 to 2012. Eur Heart J. (2017) 38:1249–58. 10.1093/eurheartj/ehw459 27997881 PMC5400048

[21] Sugimoto CR Ahn Y-Y Smith E Macaluso B Larivière V. Factors affecting sex-related reporting in medical research: a cross-disciplinary bibliometric analysis. Lancet. (2019) 393:550–9. 10.1016/S0140-6736(18)32995-7 30739690

[22] Zhang L Zhao W Sun B Huang Y Glänzel W. How scientific research reacts to international public health emergencies: a global analysis of response patterns. Scientometrics. (2020) 124:747–73. 10.1007/s11192-020-03531-4 32836522 PMC7282204

[23] Casado-Aranda L-A Sánchez-Fernández J Viedma-Del-Jesús MI. Analysis of the scientific production of the effect of COVID-19 on the environment: A bibliometric study. Environ Res. (2021) 193:110416. 10.1016/j.envres.2020.110416 33157104 PMC7607265

[24] Colavizza G Costas R Traag VA Van Eck NJ Van Leeuwen T Waltman L. A scientometric overview of CORD-19. PLoS ONE. (2021) 16:e0244839. 10.1371/journal.pone.0244839 33411846 PMC7790270

[25] Farooq RK Rehman SU Ashiq M Siddique N Ahmad S. Bibliometric analysis of coronavirus disease (COVID-19) literature published in Web of Science 2019–2020. J Fam Community Med. (2021) 28:1. 10.4103/jfcm.JFCM_332_20 33679183 PMC7927969

[26] Malik AA Butt NS Bashir MA Gilani SA. A scientometric analysis on coronaviruses research (1900–2020): time for a continuous, cooperative and global approach. J Infect Public Health. (2021) 14:311–9. 10.1016/j.jiph.2020.12.008 33618275 PMC7833583

[27] Cai X Fry CV Wagner CS. International collaboration during the COVID-19 crisis: autumn 2020 developments. Scientometrics. (2021) 126:3683–92. 10.1007/s11192-021-03873-7 33612883 PMC7882244

[28] Ebadi A Xi P Tremblay S Spencer B Pall R Wong A. Understanding the temporal evolution of COVID-19 research through machine learning and natural language processing. Scientometrics. (2021) 126:725–39. 10.1007/s11192-020-03744-7 33230352 PMC7676411

[29] Haghani M Varamini P. Temporal evolution, most influential studies and sleeping beauties of the coronavirus literature. Scientometrics. (2021) 126:7005–50. 10.1007/s11192-021-04036-4 34188334 PMC8221746

[30] Lauper K Bijlsma JW Burmester GR. Trajectories of COVID-19 information in the Annals of the rheumatic diseases: the first months of the pandemic. Ann Rheum Dis. (2021) 80:26–30. 10.1136/annrheumdis-2020-219217 33055081

[31] Bai J Li W Huang Y-M Guo Y. Bibliometric study of research and development for neglected diseases in the BRICS. Infect Dis Poverty. (2016) 5:1–10. 10.1186/s40249-016-0182-1 27595987 PMC5011792

[32] Zhongming Z Linong L Wangqiang Z Wei L. International Comparison of the UK Research Base. (2019).

[33] Nascia L Pianta M La Placa G. RIO country report 2015: Italy. Report EUR. (2016) 27850:1–89.

[34] Saxena S Paraje G Sharan P Karam G Sadana R. The 10/90 divide in mental health research: trends over a 10-year period. Br J Psychiatry. (2006) 188:81–2. 10.1192/bjp.bp.105.011221 16388075

[35] Bould M Boet S Riem N Kasanda C Sossou A Bruppacher H. National representation in the anaesthesia literature: a bibliometric analysis of highly cited anaesthesia journals. Anaesthesia. (2010) 65:799–804. 10.1111/j.1365-2044.2010.06424.x 20586744

[36] Nafade V Nash M Huddart S Pande T Gebreselassie N Lienhardt C. A bibliometric analysis of tuberculosis research, 2007–2016. PLoS ONE. (2018) 13:e0199706. 10.1371/journal.pone.0199706 29940004 PMC6016906

