# Supplementary material for: Spotlight on Early COVID-19 Research Productivity: A 1-Year Bibliometric Analysis
Source: Front Public Health. 2022 May 31;10:811885. doi: 10.3389/fpubh.2022.811885 (PMC9197383; doi:10.3389/fpubh.2022.811885)
Supplement: Supplementary file 1 [file Ttable_1.DOCX]

**SUPPLEMENTARY MATERIAL**

| **Table S1.** Scopus search terms in the title, abstract or keywords of publications on COVID-19 research and seven selected domains related to it. | |
| --- | --- |
|  | |
|  | **Search Terms** |
| **Overall literature search** |  |
| Terminology | (Covid-19) OR (SARS-COV2) OR (Coronavirus) OR (2019‐nCoV) |
| **Domain-based literature search** |  |
| Vaccination (1) | (Vaccin*) |
| Diagnosis (2) | (Diagnos*) OR (Test*) |
| Treatment (3) | (Treat*) OR (Therap*) OR (Pharma*) OR (Drug*) |
| Nutrition (4) | (Nutri*) OR (Diet*) OR (Food*) OR (Supplement*) |
| Risk factors (5) | (Risk* AND Factor*) OR (Predispos* AND Factor*) |
| Symptoms (6) | (Sign*) OR * OR (Clinical AND Present*) OR (Manifestation*) |
| Economy (7) | (Econom*) OR (Financ*) OR (Wealth*) OR (Trad*) |

|  |
| --- |
